# Robotic Exoskeleton Gait Training During Acute Stroke Inpatient Rehabilitation

**DOI:** 10.3389/fnbot.2020.581815

**Published:** 2020-10-30

**Authors:** Karen J. Nolan, Kiran K. Karunakaran, Kathleen Chervin, Michael R. Monfett, Radhika K. Bapineedu, Neil N. Jasey, Mooyeon Oh-Park

**Affiliations:** ^1^Center for Mobility and Rehabilitation Engineering, Kessler Foundation, West Orange, NJ, United States; ^2^Department of Physical Medicine and Rehabilitation, Rutgers – New Jersey Medical School (NJMS), Newark, NJ, United States; ^3^Children Specialized Hospital, Mountainside, NJ, United States; ^4^Kessler Institute for Rehabilitation, West Orange, NJ, United States; ^5^Skyline Physical Medicine and Rehabilitation, New York, NY, United States; ^6^Burke Rehabilitation Hospital, Montefiore Health System, White Plains, NY, United States

**Keywords:** rehabilitation, stroke, wearable robotics, gait, dosing, functional independence measure, exoskeleton

## Abstract

Stroke is the leading cause of severe disability in adults resulting in mobility, balance, and coordination deficits. Robotic exoskeletons (REs) for stroke rehabilitation can provide the user with consistent, high dose repetition of movement, as well as balance and stability. The goal of this intervention study is to evaluate the ability of a RE to provide high dose gait therapy and the resulting effect on functional recovery for individuals with acute stroke. The investigation included a total of 44 participants. Twenty-two participants received RE gait training during inpatient rehabilitation (RE+SOC Group), and a matched sample of 22 individuals admitted to the same inpatient rehabilitation facility-receiving conventional standard of care treatment (SOC group). The effect of RE training was quantified using total distance walked during inpatient rehabilitation and functional independence measure (FIM). The total distance walked during inpatient rehabilitation showed a significant difference between the SOC and RE+SOC groups. RE+SOC walked twice the distance as SOC during the same duration (time spent in inpatient rehabilitation) of training. In addition, the average change in motor FIM showed a significant difference between the SOC and RE+SOC groups, where the average difference in motor FIM was higher in RE+SOC compared to the SOC group. The results suggest that RE provided increased dosing of gait training without increasing the duration of training during acute stroke rehabilitation. The RE+SOC group increased their motor FIM score (change from admission to discharge) compared to SOC group, both groups were matched for admission motor FIM scores suggesting that increased dosing may have improved motor function.

## Introduction

Stroke is the leading cause of severe disability in adults, affecting approximately 15 million people each year worldwide^1^. Individuals with stroke often present with deficits in mobility, balance, and coordination, drastically limiting their activities of daily living (ADL) (Wade and Hewer, 1987; Friedman, 1990). Thus, regaining independent ambulation is a priority among stroke patients.

Current practice for motor recovery during physical therapy is based on the theory that repeated mass practice will lead to motor recovery (Lennon et al., 2001; Cooke et al., 2010). Recovery of motor function is dependent on the interrelationship between dosing, intensity (Hornby et al., 2016), and task specific practice (Krishnan et al., 2019) applied during rehabilitation. An increased number of task specific repetitions during gait training can lead to recovery of ambulatory function (Partridge et al., 2000). Recent research demonstrated that the amount of practice in the specific task is more critical than the difficulty and variations of task practice when learning new gait patterns (Krishnan et al., 2019). In particular, the same therapy with increased repetitions results in improved motor function (Byl et al., 2008; Schneider et al., 2016). Multiple studies have shown that there is a moderate relationship between the dosing and improvements in gait (Nugent et al., 1994), for example, Lang et al. (2015) have shown that intensity or dose has a moderate relationship (0.5–0.6) to the outcome in both upper and lower limb rehabilitation.

During the acute stages of recovery post-stroke, repetitive, high dose, task specific training has been found to enhance beneficial neuroplasticity and may accelerate functional recovery and the restoration of healthy gait after stroke (Langhorne et al., 1996, 2011; Kwakkel et al., 2004a,b). Research by Kwakkel et al. (2004a) has shown that greater improvements made within the first weeks post-stroke resulted in improved recovery with higher plateaus at 6 months compared to those that had delayed rehabilitation. This suggests that if recovery takes place early during the acute stage, better outcomes may be expected during the chronic stages of recovery.

Current conventional therapy has produced improvements in ambulation and motor function post-stroke. Physical therapists may not always be able to provide enough high dose, task specific repetitive gait training during the acute stages of recovery where maximum physical assistance is required (Louie and Eng, 2016). Therefore, current practices result in variable recovery of motor function, and may result in residual gait deviations and reduced functional ambulation (Kerrigan et al., 2000; Nadeau, 2014). Research is focused on increasing the dose administered to individuals with acute stroke to enhance recovery during early stages. Devices like body-weight supported treadmill were developed to increase dosing, yet high demand on the therapist still persisted especially in patients requiring maximum assistance and these devices showed limited evidence of efficacy (Nilsson et al., 2001; Mehrholz et al., 2017a). This led to focus on rehabilitative devices to assist with stepping such as gait trainers to reduce therapist effort. The gait trainers though a great step forward in providing increased consistent repetitive stepping showed marginal improvements (Peurala et al., 2009; van Nunen et al., 2015; Mehrholz et al., 2017a,b; Cho et al., 2018; Molteni et al., 2018). This could be due to reduced user initiation, or engagement of postural control for balance during standing and walking due to the body weighted system (Rojek et al., 2020). Therefore, the current research focuses on using wearable robotic exoskeletons (RE) (Eng and Tang, 2007) for overground in order to increase the task specific dosing during rehabilitation.

Wearable robotic exoskeletons (RE) are anthropomorphic mobile electromechanical devices predominantly powered bilaterally by two electric motors at the knee and hip joints (Dellon and Matsuoka, 2007; Dollar and Herr, 2008; Mohammed and Amirat, 2008). The flexion and extension at the hip and knee are actuated degrees of freedom on the device^2^ The RE provides over the ground reciprocal gait training with complete or partial assistance (Strickland, 2012). Rehabilitation with RE can provide the user with high step dose, and task specific repetition of movement, within a supported structure (improved stability) during gait training (Igo Krebs et al., 1998). The motorized movement trajectories at the hip and knee reduces the need for manual range of motion guidance. This allows physical therapists to focus on training cues and feedback to drive gait quality in a stabilized system that is providing upright support to the trunk and lower limb and ultimately reducing the number of therapists required per patient to provide effective training.

REs have been used for rehabilitation of other neurological disorders (e.g., spinal cord injury), where they provide support according to the patient's requirements (Miller et al., 2016; Tefertiller et al., 2018; Kandilakis and Sasso-Lance, 2019). But limited information is available regarding the effect of RE on functional recovery in early stage stroke rehabilitation. The objective of this study was to evaluate the ability of a RE to provide high dose gait therapy and the resulting effect on functional recovery in individuals during acute stroke. Dosing is defined as the amount of distance walked during inpatient rehabilitation.

## Methodology

### Participants

#### Robotic Exoskeleton Group

Eligible participants were admitted to an acute inpatient rehabilitation facility, diagnosed with stroke (<6 months), between the ages 18 and 82 years and had to physically fit into the RE device (height 152.4–177.8 cm; weight <99.7 kg; hip width 35–46″). All participants' lower limbs had: (1) no history of injury or pathology (unrelated to their stroke) within the last 90 days; (2) joint range of motion (ROM) within normal functional limits for ambulation; (3) no lower limb joint contracture or spasticity that limits ROM during ambulation; (4) sufficient strength of the contralateral limb to use an assistive device for ambulation; (5) ability to communicate and follow one step instructions at a level consistent with standard motor rehabilitation; (6) upper body strength to balance with a walker or cane; (7) no medical issues that precludes full weight bearing and ambulation; (8) no skin issues that would prevent wearing the device; (9) stable blood pressure, no diagnosis of persistent orthostatic hypotension, uncontrolled hypertension, coronary artery disease and (10) able to tolerate upright standing for up to 30 min with assistance; (11) time since injury <50 days; (12) RE participants received at least 3 days of RE training; (13) length of stay <50 days. Exclusion criteria were contracture of joints (hip, knee, and ankle) that would prohibit a healthy range of motion without pain as well as fitting the RE. Additional exclusion criteria were cardiopulmonary or other medical conditions that prohibit intensive gait training. Out of the 27 participants post-stroke who received RE gait training during inpatient rehabilitation (RE+SOC Group), five were excluded from further analysis as they could not be matched to control group based on match criteria explained below. The investigation was approved by the Kessler Foundation Institutional Review Board and all participants consented to participate in the study.

#### Matched Participants (Standard of Care) Control Group

Twenty-two patients post-stroke participated in the intervention group with RE gait training (RE+SOC Group). The matched control group was identified retrospectively through eRehabData® an inpatient rehabilitation outcomes system and data was exported from the Inpatient Rehabilitation Facility (IRF) during the same time period of the investigation. All patients in RE+SOC group and control group (matched sample SOC group) were recruited from the same facility. Using custom Matlab (The Mathworks Inc., Natick, MA, USA) programming each participant (*n* = 22) who received at least 3 visits of RE gait training was matched for age (within 6 years), length of stay (within 2 days), admission FIM motor score (within 4 points), gender, and hemiparetic side. The detailed demographics and clinical characteristics of the RE+SOC group and matched control group are presented in Table 1.

**Table 1:** Participant demographics (mean ± standard error).

**Group**	**RE+SOC Group**	**SOC Group (Matched Sample)**
**Age (Years)**	59.86 ± 1.99	59.41 ± 2.23
**Height (m)**	1.72 ± 0.02	1.73 ± 0.02
**Weight (Kg)**	79.04± 3.26	82.9 ± 3.38
**LOS (Days)**	30.23 ± 1.58	29.63 ± 1.63
**Admission Motor FIM**	25.72 ± 1.31	25.45 ± 1.39
**Discharge Motor FIM**	54.05 ± 2.03	48.82 ± 2.35
**Admission Walk FIM**	1.05± 0.05	1.27 ± 0.149
**Discharge Walk FIM**	4.27 ± 0.24	4.09 ± 0.3
**Gender**	18 males, 4 females	18 males, 4 females
**Affected Side**	14 w/right hemiplegia	14 w/right hemiplegia
**Time Since Injury (Days)**	10.0 ± 2.5	12.6 ± 2.4
**RE Sessions**	3.72 ± 0.18 (range 3-5)	-
**PT Sessions**	21.14 ±1.23	19.5 ± 1.2

*Length of Stay (LOS)—Calculated as the number of calendar days from the date of admission to the IRF to date of discharge from the IRF*.

*Admission Motor Functional Independence Measure (FIM)—Calculated as the total score for all motor sections of the FIM, as measured at admission to the IRF*.

*Discharge Motor Functional Independence Measure (FIM)—Calculated as the total score for all motor sections of the FIM, as measured at discharge from the IRF*.

*Time Since Injury—Calculated as the number of calendar days from the date of injury to the date of admission to the IRF*.

*RE Sessions—The average number of intervention sessions with the RE during their length of stay in the IRF*.

*Physical Therapy (PT) Sessions—The average number of physical therapy sessions throughout their length of stay at the IRF, including RE sessions*.

The equivalence between the groups was established using Cohen's d effect size using number of physical therapy sessions, distance walked during initial evaluation, and motor FIM score at admission. Cohen's d effect size was small for distance walked during initial evaluation (Cohen's d effect size = 0.13, Mean_SOC_ = 3.74 m, SD_SOC_ = 7.0203 Mean_RE+SOC_ = 3.05 m, SD_RE+SOC_ = 2.74) and motor FIM score at admission (Cohen's d effect size = 0.2653, Mean_SOC_ = 25.5, SD_SOC_= 6.5, Mean_RE+SOC_ = 25.7, SD_RE+SOC_ = 6.2). The small effect size signifies that there is no difference between the groups in terms of distance walked during initial evaluation and motor FIM score at admission. This establishes that both groups were similar at admission. Cohen's d effect size was small for number of physical therapy sessions (Cohen's d effect size = 0.29, Mean_SOC_ = 19.5, SD_SOC_ = 5.62, Mean_RE+SOC_ = 21.14, SD_RE+SOC_ = 5.76). The number of physical therapy sessions was determined by the physical therapist and were driven by the patient's recovery progression.

### Robotic Exoskeleton (RE) Device

**Figure 1:**
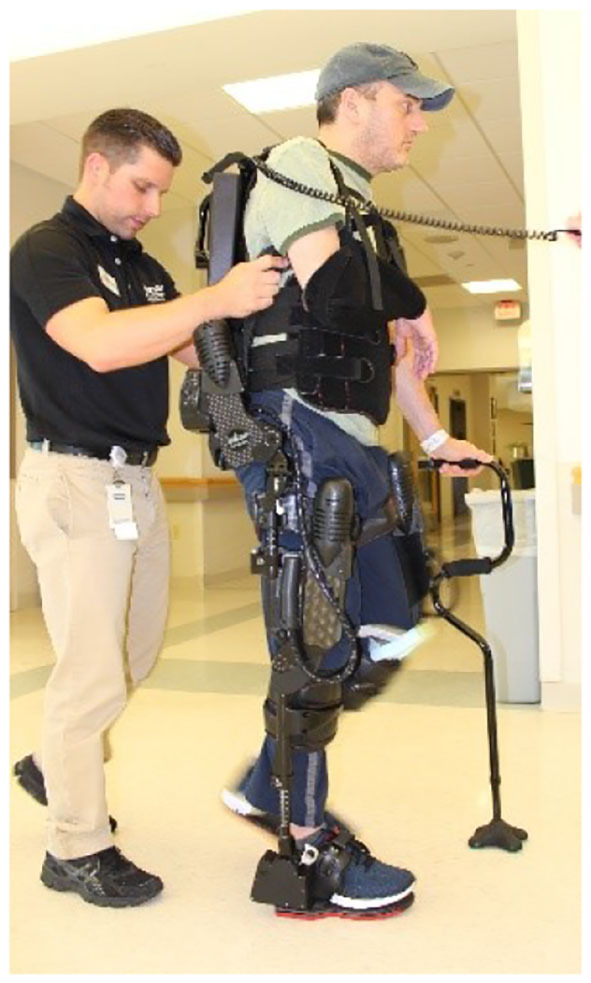
Robotic Exoskeleton (RE) gait training with a physical therapist.

Robotic gait training was provided to participants in the RE+SOC Group during stroke rehabilitation at an inpatient rehabilitation hospital through a commercially available, FDA class 2 approved [510(k) number is K143690] exoskeleton (Ekso GT™, Ekso Bionics, Inc. Richmond, CA, USA). The RE is intended for overground gait rehabilitation under the guidance of a licensed physical therapist. The device is individually programmed to provide motor assistance to patients by driving their lower extremity through a repetitive predefined gait trajectory for strength and endurance training. The device was attached to the user, with backpack style shoulder harness, a torso brace, affixed to the legs with upper thigh straps and shin guards on the shank, and a secure foot binding (Figure 1). The RE includes two powered joints (hip and knee) which provide bilateral angular motion in sagittal plane (hip flexion/extension and knee flexion) and a passively sprung ankle joint with adjustable stiffness that provides resistance in the sagittal plane (dorsiflexion and plantarflexion). The actuated ROM at the hip is −20° to 135° and the actuated range for the knee is 0° to 120°. The range of motion provided at the ankle is from −10° to 20° dorsiflexion. Additional ROM is provided to assist with functions such as standing and sitting.

### Experimental Procedures

#### RE Gait Training

The RE was used for gait training with variable bilateral assistance during inpatient rehabilitation. Training with the RE was completed during standard therapy at least 3 times during their inpatient rehabilitation stay. RE gait training was administered during scheduled physical therapy sessions to avoid patients in the RE+SOC group receiving additional therapy during their inpatient stay. A licensed physical therapist directed all RE gait training sessions and adjusted the ambulation assistance according to every individual participant's progress. Participants received conventional physical therapy session (see Standard of Care Gait Training) when they were not trained with the RE.

The physical therapist adjusted the walking pattern (i.e., step speed and length), and the robotic assistance to facilitate therapeutic progression. The treating physical therapist utilized variable assistance (changes to the level of assistance provided by the RE) to account for asymmetrical walking function or assist with weakness (post-stroke hemiplegia). The control technique (steps) of the robot was triggered by patient movement initiating a first step. For example, participants would shift their center of mass laterally, while offloading the back limb during toe-off in preparation for the next step (RE step mode: ProStep+) or participants shifting their COM anterior and laterally (ProStep). When appropriate, steps were triggered by the physical therapist to start the walking sequence.

#### Standard of Care Gait Training

A licensed physical therapist administered all standard of care therapy sessions. Each session included gait training, standing tolerance, balance, endurance, and pre-gait activities. Sessions emphasized weight bearing through the affected side to promote sensory awareness and motor recovery through upright activities and walking. Participants completed known walking distances during gait training and this clinical information was recorded in the medical chart by the physical therapist.

Both SOC and RE+SOC groups received the same amount of therapy time overall and walking distances were individualized by a licensed physical therapist based on patient progression. Each gait training session included at least 45 min of therapy.

#### Outcome Measures and Statistical Analysis

Walking distance is a reliable objective outcome measure after stroke and is a key indicator of functional ambulation (Bohannon et al., 1991). Distance walked was collected during each RE and physical therapy sessions for the RE+SOC and during physical therapy sessions for the SOC group. Total distance walked was used to quantify the amount of dosing during their inpatient rehabilitation. Distances walked during physical therapy were extracted from the medical chart and distances walked during RE sessions were calculated ([RE steps x step length from Ekso bionics settings]/12 [divided by twelve to convert from English to metric system]) from the RE software. Outcome measures included: (1) Total Distance Walked; (2) Total Distance Walked during conventional physical therapy sessions for the RE+SOC and SOC group (excludes any sessions in the RE); (3) Maximum Distance Walked; and (4) Distance During Each Session (Table 2).

**Table 2:** Distance outcome measures.

**Outcome Measure**	**Description**
Total Distance Walked (m)	• RE+SOC = Sum of the total distance walked in all RE and physical therapy sessions throughout the LOS at the IRF • SOC = Sum of the total distance walked in all physical therapy sessions throughout the LOS at the IRF
Total Distance Walked during Physical Therapy (m)	Sum of the total distance walked in all conventional physical therapy sessions throughout the LOS at the IRF, this excludes any sessions in the RE
Average Total Distance (m)	Total distance walked during the LOS at IRF by all participants/ number of participants.
Maximum Distance Walked (m)	• RE+SOC = Maximum distance walked during a single physical therapy session after the start of RE gait training • SOC = Maximum distance walked during a single physical therapy session throughout the LOS at the IRF
Average Maximum Distance (m)	Maximum distance walked during the LOS at IRF by all participants/ number of participants.
Distance During Each Session (m)	Sum of the total distance walked in all physical therapy sessions (including RE sessions)/Number of physical therapy sessions
Average Distance During Each Session (m)	Sum of Distance Walked per Session by all participants/ number of participants

The FIM was collected at admission and discharge for all participants at the same inpatient rehabilitation facility (IRF). The FIM is an 18 items scale that includes 13 motor tasks and 5 cognitive tasks rated on a 7-point ordinal scale from complete dependence to complete independence. A score of seven indicates that you are completely independent in that particular activity and a score of one means that you require total assistance for the activity. The 13 FIM motor items range from 13-91 points and rates an individual's ability to perform motor activities of daily living independently (Imada et al., 2014). The FIM motor score at admission and discharge, and the length of stay for each participant in the RE+SOC and SOC groups were used to calculate Motor FIM outcome variables: (1) Motor FIM change (MFC); and (2) Motor FIM Efficiency (MFE) (Table 3). The locomotion domain, specifically the walk component of the Motor FIM was further evaluated as an outcome for the RE+SOC and SOC groups. The Motor FIM Walk score at admission and discharge, and the length of stay of each participant in the RE+SOC and SOC groups were used to calculate Walk FIM outcome variables: (1) Walk FIM change (WFC); and (2) Walk FIM Efficiency. The Motor FIM change and Walk FIM change were considered a more reliable metric of recovery compared to the absolute values. The change score evaluated progression with respect to impairment at admission.

**Table 3:** FIM outcome measures.

**Outcome measure**	**Description**
Motor FIM Change (MFC)	Admission Motor FIM-Discharge Motor FIM
Average Motor FIM Change	Average Motor FIM Change = Sum of MFC of all participants/ Number of participants
Motor FIM Efficiency (MFE)	(Admission Motor FIM-Discharge Motor FIM)/length of stay
Average Motor FIM Efficiency	Sum of MFE of all participants/ number of participants
Walk FIM Change (WFC)	Admission Walk FIM-Discharge Walk FIM
Average Walk FIM Change	Average Walk FIM Change = Sum of WFC of all participants/ Number of participants
Walk FIM Efficiency (WFE)	(Admission Walk FIM-Discharge Walk FIM)/length of stay
Average Walk FIM Efficiency	Sum of WFE of all participants/Number of participants

Demographic data were analyzed using descriptive statistics. Independent sample *t*-tests were performed to determine the difference between the two groups (RE+SOC and SOC groups) as Kolmogorov-Smirnov test and Levene's test for equality of variance showed that data was normal (*p* > 0.05) and of equal variance (*p* > 0.05), respectively for selected outcome variables: (1) Motor FIM Change; (2) Walk FIM Change; (3) Walk FIM Efficiency; (4) Maximum Distance; (5) Motor FIM Efficiency; and (6) Total Distance during conventional training. Mann-Whitney *U*-Test was performed to determine the difference between the two groups (RE+SOC and SOC groups) as the Kolmogorov-Smirnov test showed that data was not normal (*p* < 0.05) for Distance Walked per session and Total Distance. Spearman's Correlation was used to determine the relationship between Motor FIM Change vs. Total Distance since total distance data was not normal. Pearson's r Correlation was also used to determine the relationship between Motor FIM Change and number of RE sessions.

## Results

### Distance

**Figure 2:**
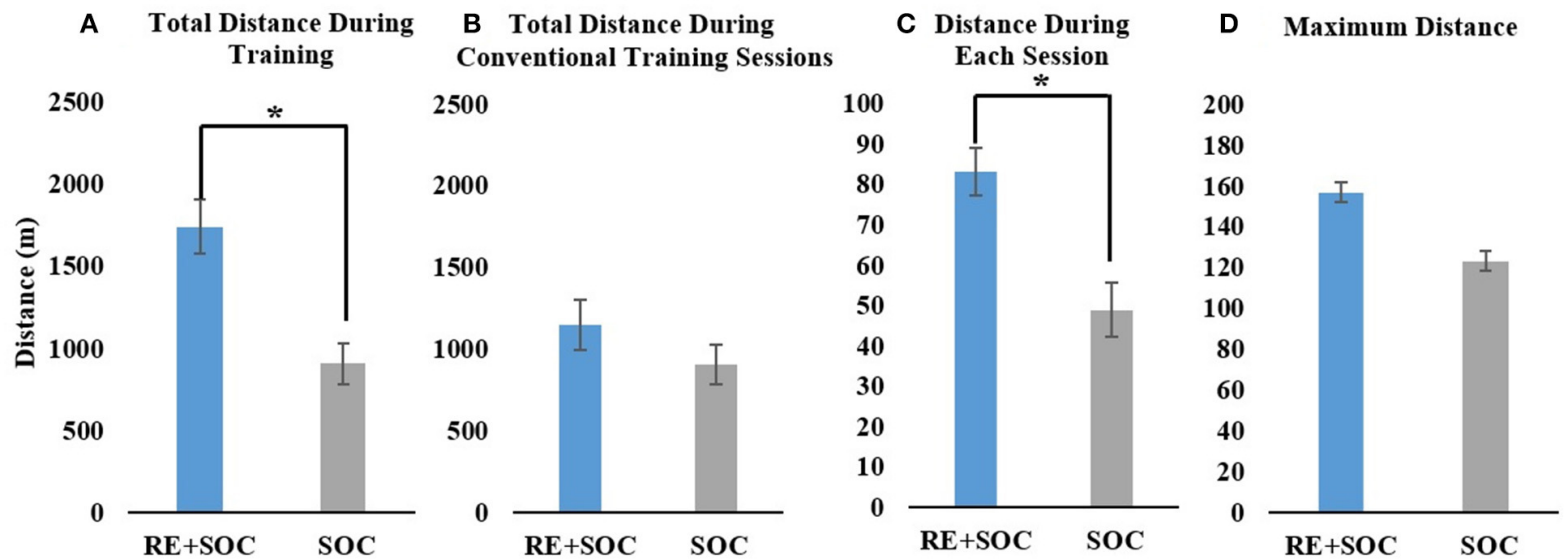
**(A)** The total distance walked during inpatient rehabilitation for the RE+SOC (includes sessions with the RE and during standard of care physical therapy) and SOC group (includes sessions during standard of care physical therapy); **(B)** The total distance walked during conventional physical therapy (standard of care physical therapy, excludes any sessions with the RE) during inpatient rehabilitation for the RE+SOC and SOC groups; **(C)** The average distance walked during each physical therapy session; and **(D)** The maximum distance walked during conventional physical therapy for the LOS. In case of the RE+SOC group, the average maximum distance was determined as the maximum distance after the first RE training. All data are presented as mean ± standard error. *Significance of *p* < 0.05.

Average total distance during inpatient rehabilitation showed a significant difference (*p* < 0.05, Mean_SOC =_ 906.96, SE_SOC =_ 123.42.8, Mean_RE+SOC =_ 1742.7, SE_RE+SOC =_ 163.3) between the SOC and RE+SOC group. RE+SOC group walked twice the distance as the SOC group during the same the same duration (time spent in inpatient rehabilitation) of training (Figure 2A). Average total distance during PT training did not show a significant difference (*p* > 0.05, Mean_SOC =_ 906.96, SE_SOC =_ 123.42, Mean_RE+SOC =_ 1147.1, SE_RE+SOC =_ 152.1) between the SOC and RE+SOC group. The RE+SOC and SOC group received comparable training in their SOC sessions (Figure 2B) though distance walked by the RE+SOC was slightly more than the SOC group. Average distance during each session walked showed a significant difference (*p* < 0.05, Mean_SOC =_ 48.99, SE_SOC =_ 6.7, Mean_RE+SOC =_ 83.2, SE_RE+SOC =_ 5.8) between the SOC and RE+SOC group (Figure 2C). Average maximum distance in a session did not show a significant difference (*p* > 0.05, Mean_SOC =_ 123.33, SE_SOC_= 16.03, Mean_RE+SOC =_ 157.03, SE_RE+SOC =_ 16.41) between the SOC and RE+SOC group. Though the maximum distance in a session walked by the RE+SOC group was more than the SOC group (Figure 2D).

### FIM and FIM Efficiency

**Figure 3:**
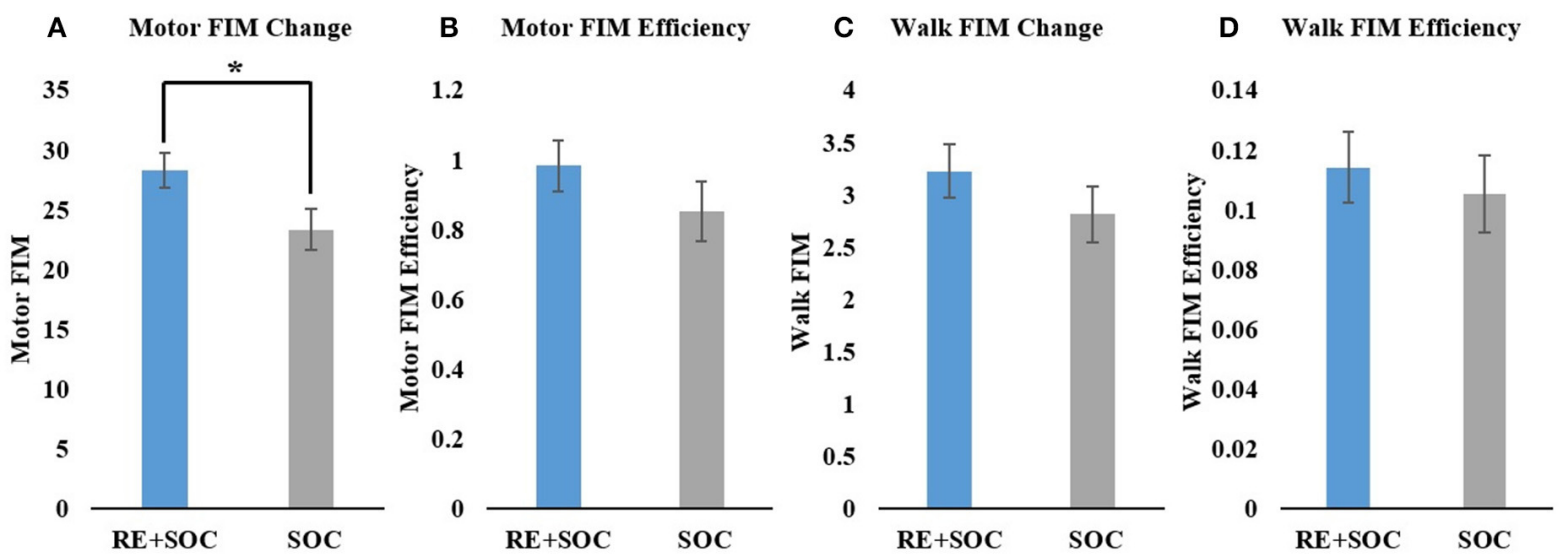
**(A)** Change in Motor FIM from admission to discharge; **(B)** Motor FIM efficiency, **(C)** Change in Motor FIM walk component from admission to discharge; and **(D)** Motor FIM walk component efficiency. All data are presented as mean ± standard error. *Significance of *p* < 0.05.

Average change in motor FIM showed a significant difference (*p* < 0.05, Mean_SOC_ = 23.36, SE_SOC_ = 1.74, Mean_RE+SOC_ = 28.3, SE_RE+SOC_ = 1.5) between the SOC and RE+SOC group where the average difference in motor FIM was higher in RE+SOC compared to the SOC group (Figure 3A). Average motor FIM efficiency did not show a significant difference (*p* > 0.05, Mean_SOC_ = 0.85, SE_SOC_ = 0.086, Mean_RE+SOC =_ 0.98, SE_RE+SOC_ = 0.07) between the SOC and RE+SOC group. Average motor FIM efficiency was higher in RE+SOC compared to the SOC group (Figure 3B).

In order to further understand the impact of RE on gait, we evaluated the specific walking components of motor FIM. Average difference in Walk FIM did not show a significant difference (*p* > 0.05, Mean_SOC_ = 2.82, SE_SOC_ = 0.27, Mean_RE+SOC_ = 3.2, SE_RE+SOC_ = 0.25) between the SOC and RE+SOC group, though the average difference in Walk FIM increased in RE+SOC compared to the SOC group (Figure 3C). Average difference in Walk FIM efficiency did not show a significant difference (*p* > 0.05, Mean_SOC_ = 0.105, SE_SOC_ = 0.01, Mean_RE+SOC_ = 0.114, SE_RE+SOC_ = 0.01) between the SOC and RE+SOC group, although the average difference in Walk FIM efficiency was higher in RE+SOC compared to the SOC group (Figure 3D).

### Correlation Between Total Distance and Average Difference in Motor FIM

**Figure 4:**
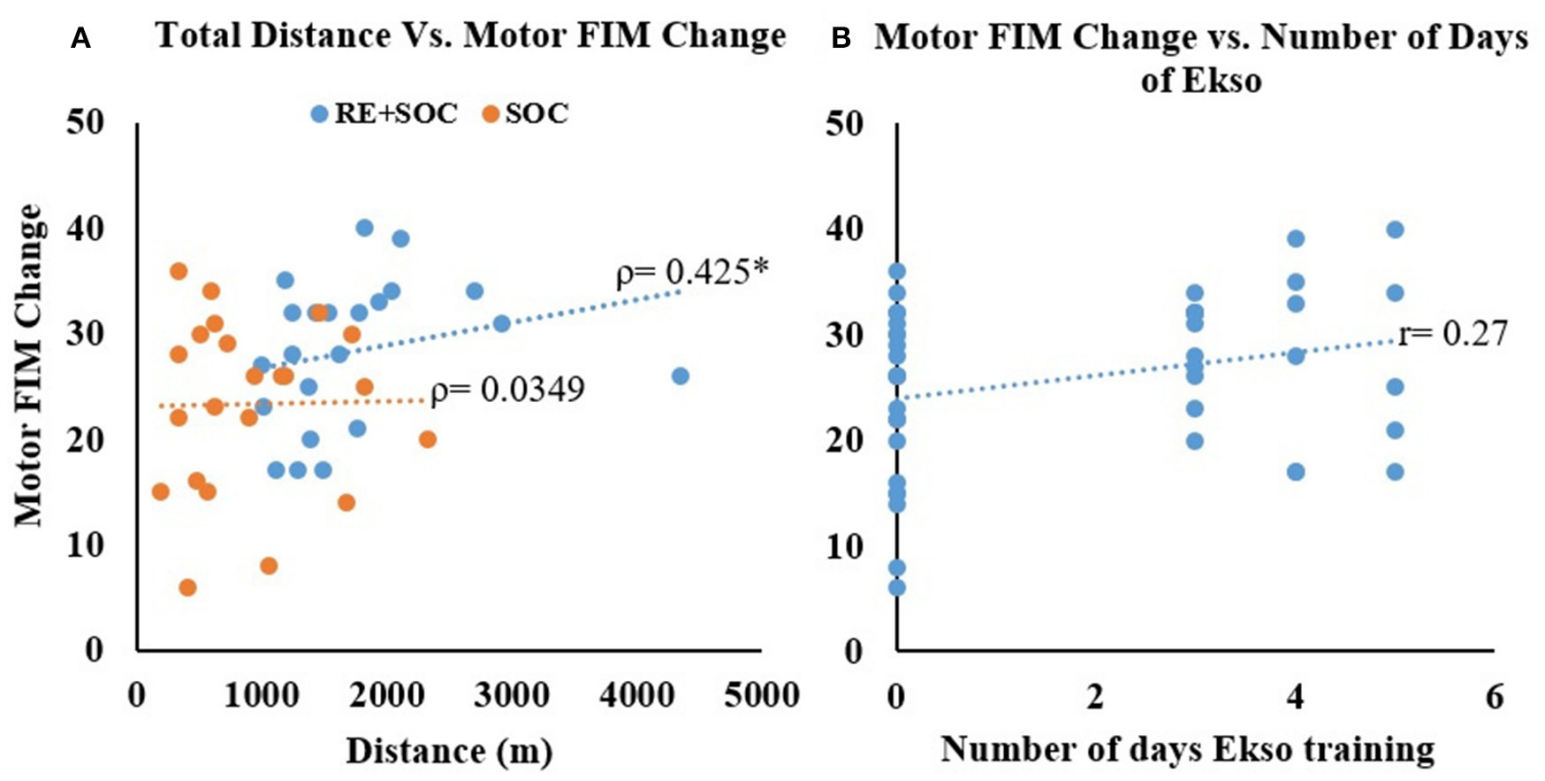
**(A)** The relationship between total distance walked and change in motor FIM from admission to discharge. **(B)** The relationship between number of days of RE training and change in motor FIM from admission to discharge for all participants. *Significance of *p* < 0.05.

No Correlation was observed between total distance and difference in Motor FIM without RE (Spearman's rho = 0.039, *p* = 0.862) but with RE training the significant correlation between total distance and difference in Motor FIM—(Spearman's rho = 0.425, *p* = 0.048) was observed (Figure 4A). There was also small increase in Pearson's r correlation between number of days of Ekso training and change in motor FIM though was not statistically significant (Figure 4B).

## Discussion

Individuals with acute stroke have significant ambulatory deficits resulting in reduced mobility and activities of daily living (Wade and Hewer, 1987; Friedman, 1990). Current research is focused on improving ambulation using robotic lower extremity exoskeletons, which can provide high dose repetitive training. In this study, the differences in the motor rehabilitation dosing and its resulting effect on functional recovery was evaluated between RE+SOC and SOC in individuals with acute stroke.

Participants with acute stroke had similar motor FIM scores (Table 1) at admission indicating similar levels of impairment. They may have been at different stages in their rehabilitation requiring different levels of physical assistance for ambulation. RE can provide assistance as required by the user while proving consistent repetitive practice to drive recovery. Total distance walked was used as a measure of dosing during their inpatient rehabilitation. The RE+SOC group walked twice the distance compared to SOC group during inpatient rehabilitation as measured by the total distance walked (Figure 2A). The distance per session was also significantly higher in RE+SOC group compared to SOC group (Figure 2C). The RE+SOC group received the same duration of training (time spent in inpatient rehabilitation training session) and similar dosing during conventional physical therapy sessions as the SOC group (Figure 2B). Thus, RE provided increased dosing of gait training without increasing the duration of training. This suggests that RE could enhance dosing during motor rehabilitation especially for individuals with acute stroke who require maximum assistance.

Current rehabilitation theories are based on the concept of neuroplasticity, which states that repeated high dose task-specific practice could lead to recovery of function (Lennon et al., 2001; Cooke et al., 2010). Our results are in accordance with this theory, where increased walking dose due to RE training resulted in increased maximum distance walked for the RE+SOC group in a single session as compared to SOC group (Figure 2D). This is an indicator of functional ambulation recovery possibly suggesting that RE training enhances functional recovery. Increased walking distance is associated with increased community ambulation and participation leading to improved quality of life (An et al., 2015).

Recent research demonstrated that the amount of practice in the specific task is critical for gait recovery (Krishnan et al., 2019). Multiple studies suggest a moderate relationship between frequency of repetitions and improvements in motor function (Nugent et al., 1994). In particular, studies have shown that the same therapy, with increased repetitions, produced improved function (Byl et al., 2008; Schneider et al., 2016). Lang et al. (2015) demonstrated that intensity or dose has a moderate relationship (0.5–0.6) to the outcome in both upper limb and gait rehabilitation. Our results are in accordance with previous research that increased dosing results in improved motor function. The RE+SOC group increased their motor FIM score (change from admission to discharge) and Motor FIM efficiency compared to SOC group though both groups were matched for admission motor FIM scores (Figure 3A). Motor FIM change was significant and was representative of the cumulative change per day (Motor FIM efficiency, Figure 3B). This could indicate that adding dosing through RE could improve the motor recovery trajectory long term. In addition, there was an increase in Walk FIM (Figure 3C), which indicates that the motor FIM were in the locomotor domain.

In order to understand the underlying mechanism of functional recovery, we explored the relationship between increased dose and functional improvements (Figure 4). The relationship between total distance walked and difference in Motor FIM (Figure 4A) improved when RE training was added to the inpatient stroke rehabilitation program. However, there was no correlation between total distance walked and difference in Motor FIM in the SOC group. This may be due to the SOC group not receiving sufficient dosage to induce a change in motor FIM. These results indicate that increased dosing from RE training could be contributing to improved functional outcomes, such as improved motor FIM. This is evidenced by Figure 4B, which shows that there is improved motor FIM change with increased use of RE. Though the change in FIM is small, the impact of even small incremental changes on ADL and QOL could be substantial, especially for individuals with acute Stroke (Imada et al., 2014). Our results are in accordance with previous research demonstrating that wearable REs for overground gait training have the ability to improve functional ambulation in sub-acute and chronic patients post-stroke (Molteni et al., 2017). Overground REs require active participation and the patient is responsible for maintaining trunk and balance control (Molteni et al., 2017, 2018). Increased dosing in combination with active participation will promote brain plasticity and connectivity re-modulation that are specifically entrained by the robotic device, as compared to conventional gait training (Androwis et al., 2018; Calabrò et al., 2018; Molteni et al., 2018).

Our sample of 22 participants did not have any secondary complications or falls due to the RE. The RE training in this preliminary study was restricted to 3–5 sessions during inpatient rehabilitation, and the participants (*n* = 22) continued to receive their SOC for stroke rehabilitation. One of the major limitations of the study was not having a separate group for RE-only training. Consequently, future studies should standardize the amount of RE training matched to the control group.

Previous research showed that greater improvements within the first weeks post-stroke resulted in higher plateaus at 6 months than improvements occurring to those that had delayed rehabilitation (Kwakkel et al., 2004a). This suggests that if recovery takes place early post-stroke, better outcomes may be expected during the chronic stages of recovery. Our results suggest that an RE can provide the crucial high dose task specific training during acute inpatient rehabilitation, and may aid in early recovery onset.

## Data Availability Statement

The datasets presented in this article are not readily available because current Kessler Institutional Review Board approval does not include data sharing with external institutions. Requests to access the datasets should be directed to knolan@kesslerfoundation.org.

## Ethics Statement

The studies involving human participants were reviewed and approved by Kessler Foundation Institutional Review Board. The patients/participants provided their written informed consent to participate in this study. Written informed consent was obtained from the individuals for the publication of identifiable images included in this article (Figure 1).

## Author Contributions

KN and MO-P designed the study. KN and KK analyzed the data. KN, KK, and MO-P drafted and finalized the manuscript. KN, KC, MM, RB, NJ, and MO-P assisted with data collection. All authors contributed to the article and approved the submitted version.

## Conflict of Interest

The authors declare that the research was conducted in the absence of any commercial or financial relationships that could be construed as a potential conflict of interest.

## Footnotes

^1^*Stroke Statistics*. Available online at: http://www.strokecenter.org/patients/about-stroke/stroke-statistics/ (accessed August 5, 2018).

^2^*Ekso Bionics*. Available online at: https://eksobionics.com/.

## Abbreviations

ADL: activities of daily living
FIM: Functional Independence Measure
IRF: inpatient rehabilitation facility
LOS: length of stay
RE: robotic exoskeletons
ROM: range of motion
SOC: standard of care
QOL: quality of life.
